# Factors Associated with Anatomic Failure and Hole Reopening after Macular Hole Surgery

**DOI:** 10.1155/2021/7861180

**Published:** 2021-12-07

**Authors:** Yutong Li, Siyan Jin, Lijun Shi, Hecong Qin, Jinsong Zhao

**Affiliations:** Department of Ophthalmology, The Second Hospital of Jilin University, 4026 Yatai Street, Changchun City 130000, China

## Abstract

A macular hole (MH), particularly an idiopathic macular hole (IMH), is a common cause of central vision loss. Risk factors for nonidiopathic MH include high myopia, cystoid macular edema, inflammation, and trauma. MH is primarily diagnosed using slit-lamp microscopy and optical coherence tomography (OCT). Half of the patients with stage I MHs are treated conservatively and may show spontaneous resolution. The main treatment methods for MHs currently include vitrectomy and stripping of the internal limiting membrane (ILM). However, in some patients, surgery does not lead to anatomical closure. In this review, we summarize the factors influencing the anatomical closure of MHs and analyze the potential underlying mechanisms.

## 1. Introduction

A macular hole (MH) is a vitreoretinal interface disease characterized by a retinal defect in the center of the macula. MHs are detected in people with highly myopic eyes or those who have ocular trauma. However, in a large majority of cases, MHs are idiopathic. The diagnosis and treatment plans for MH have advanced rapidly in the last two decades. Optical coherence tomography (OCT) is used in the diagnosis and follow-up post-treatment. Furthermore, surgery allows for a MH closure rate of >90%.

Pars plana vitrectomy (PPV) has been used for more than a decade to treat MH. PPV is a surgical technique to relieve traction by removal of the vitreous body. Other treatments for MH include internal limited membrane peeling (ILMP), tamponade, and postoperative positioning. However, some MHs may remain open postoperatively. In this review, we aimed to summarize the factors associated with anatomic failure and reopening after MH surgery. Our findings may help doctors determine the presence of risk factors for postoperative anatomic failure and develop appropriate treatment methods. It may also encourage further improvements in surgical techniques aiming to increase the hole closure rate. Based on the etiologies and surgical processes, we summarized the factors hindering the closure or causing MH recurrence by classifying them into preoperative, intraoperative, and postoperative factors.

## 2. Preoperative Factors

### 2.1. Duration

The effect of disease duration on MH closure is controversial. Brockmann et al. [1] divided patients with idiopathic macular hole (IMH) (160 eyes) into five groups depending on disease duration: <1 month, 1–3 months, 4–6 months, 7–12 months, and >12 months. Statistical analysis showed that the closure rate of the hole was not related to disease duration. However, disease duration remains a widely recognized risk factor affecting the anatomical healing of an IMH. A prospective cohort study by Essex et al. [2] involving 2,456 eyes with IMH, reported that duration of >9 months reduced the postoperative closure rate of IMH. Thompson et al. [3] speculated that an increase in the duration might lead to an increase in the hole diameter along with the appearance of the epiretinal membranes (ERMs), which expand the hole and impede closure. Additionally, prolonged exposure causes progressive damage to the pigment epithelium and photoreceptor cells. Furthermore, as the duration increases, the microenvironment surrounding the MH changes, fluid accumulates in the subretinal cavity, and the retina stiffens. This leads to a decline in retinal compliance and a failure in the hole closure.

### 2.2. Various Parameters

Gass [4] classified IMH into four stages currently used worldwide. IMH stage is a good predictor of postoperative anatomical closure. Patients with stage ≤III IMH reportedly have a higher closure rate than those with stage IV IMH [5, 6].

The applications and advancement of OCT technology contribute to postoperative result prediction based on hole parameters. The height (*H*), reflecting the vertical traction force for the macular area, represents the distance from the retinal pigment epithelium (RPE) to the innermost aspect of the MH. The minimum diameter (MIN) represents the minimum distance between the broken ends of the neuroepithelium. In contrast, the base diameter (BASE) represents the diameter of the MH at the level of the RPE. Ip et al. [7] proposed that the closure rate depending on the MIN is ascending in the following order: >500 *μ*m, >400 *μ*m, and <400 *μ*m. Among these parameters, Wakely et al. [8] considered BASE the most useful variable because it was most associated with anatomical results. The key BASE value for predicting anatomical success is 747.5 *μ*m. According to their receiver operating characteristic curve corresponding to anatomical success and BASE, 747.5 *μ*m was used as the differential value for predicting anatomical outcomes, with 100% specificity and 76.2% sensitivity. Furthermore, a BASE range of 721.0–747.5 *μ*m may retain the specificity of 100% and sensitivity of >70%. Thus, both MIN and BASE may reflect the tangential traction and have negative correlations with MH closure.

In 2004, Kusuhara et al. [9] introduced the macular hole index (MHI), defined as the H-to-BASE ratio. The results showed that the larger the MHI values, the smaller the change in the morphology of the macular fovea and the higher the closure rates. Subsequent studies have also demonstrated that the MHI is highly correlated with postoperative anatomical outcomes and is a good predictor of anatomical success [10].

**Figure 1:**
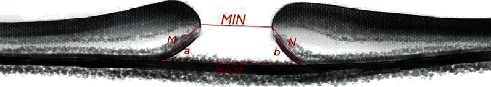
HFF (hole form factor) = (M + N)/BASE (base diameter); MHCI (macular hole closure index) = (a + b)/BASE.

The hole form factor (HFF) [11] refers to the ratio of the sum of the same-side distances between the ends of the MIN and BASE to the diameter of the BASE (Figure 1). It reflects the relationship between the sum of the lengths of the separated photoreceptors on both sides and BASE. The exposed RPE layer is completely covered postoperatively when HFF is > 1; therefore, holes heal successfully. Ullrich et al. found that eyes with a HFF of >0.9 show a markedly higher closure rate than those with a HFF of <0.5 [11].

Ruiz-Moreno et al. [10] introduced the MH tractional hole index (THI), that is, the ratio of maximum H to MIN. They also introduced the MH diameter hole index (DHI), namely, the ratio of MIN to BASE. A MIN >311 *μ*m and THI >1.41 indicate a good prognosis; however, the DHI and prognosis are not strongly correlated. The THI represents the relationship between the two tractions responsible for the MH. The larger the THI value, the smaller the morphological change in the macular fovea; this condition favors hole closure [12]. However, some scholars believe that the MH diameter is a better predictor of hole closure than the parameters discussed above [8, 13].

In 2016, Liu and Zhao [14] presented the macular hole closure index (MHCI), calculated as the ratio of the sum of the curve lengths of the detached photoreceptor arms to BASE. The ability of detached photoreceptors to cover the exposed RPE layer postoperatively contributes markedly to the hole closure. In this study, MH closure was divided into three types: bridge-like closure, good closure, and poor or no closure. The larger the MHCI, the easier it is to achieve a bridge-like closure. When the MHCI is too small, the possibility of poor closure is high in the exposed RPE layer. When the MHCI ranges from 0.7 to 1, good closure with normal foveal morphology may be achieved.

### 2.3. High Myopia-Related Factors

A highly myopic MH is a significant type of MH. The main pathological changes in high myopia include axial elongation and posterior staphyloma, as well as tangential forces resulting from the insufficient elasticity of the ILM and chorioretinal atrophy. Numerous researchers reported that the closure rate of highly myopic MHs is lower than that of IMHs.

#### 2.3.1. Axial Length

Before the emergence of OCT, no consensus was established on the relationship between axial elongation and poor prognosis of MH. In 2001, Patel et al. [15] reported that the closure rate of MH was relatively low in patients with high myopia. Conversely, in case-control studies by Sulkes et al. [16] and Kobayashi et al. [17], no relationship was observed between the axial length and the anatomical success rate. However, most of the above studies used biomicroscopy to determine whether the MH was closed; hence, their results were inaccurate.

In 2011, with the help of OCT, Suda et al. [18] found that an axial length of ≥30.0 mm was a risk factor for failure of anatomic closure. They divided patients with MH into three groups according to an axial length: group 1, axial length ≥30.0 mm; group 2, 30 mm > axial length ≥26.0 mm; and group 3, axial length <26.0 mm. Among them, the postoperative closure rate of group 1 was significantly lower than that of the latter two groups; however, no statistically significant differences were observed between groups 2 and 3. Suda et al. reported that the rate of anatomical success decreased while the axial length increased. This may be attributed to the fact that the retina cannot change along with the elongation of the ocular axis. Consequently, a vertical traction force, acting on the surface of the retina, is produced [19, 20]. The posterior retina may also gradually lose its elasticity in the process of stretching with the growth of the ocular axis, thus affecting the hole closure [21]. Furthermore, in eyes with a long axial length, the operation is highly complicated. Therefore, there may be a remnant vitreous body and internal limiting membrane (ILM), potentially hindering the closure of the hiatus [21]. In 2012, Wu and Kung [19] conducted a study on 42 eyes. This study showed that, in the high myopia group, posterior staphyloma or an axial length ≥30.0 mm existed in all eyes with an unclosed hiatus. Nadal et al. [22] highlighted that eyes with an axial length ≥30.0 mm had a significantly lower closure rate than those with an axial length <30.0 mm. An axial length of 30 mm is considered essential for evaluating the healing of postoperative hiatus.

#### 2.3.2. Posterior Staphyloma

Due to the elongated axial length and outpouching of the posterior fundus found in high myopia, the sclera expands progressively, forming a posterior staphyloma [23], which mainly occurs around the optic disc.

Although the effect of posterior staphyloma on the prognosis of MH remains unclear, numerous researchers believe that the presence of posterior staphyloma may cause additional anteroposterior traction and hinder MH closure.

In 2003, Ikuno et al. [24] studied 16 cases of MHs with high myopia and retinal detachment using OCT. They found that the low closure rate of MH may be associated with the pathological changes of myopia, including the axial elongation and formation of posterior staphyloma. Yasushi et al. reported that the retina was too short to cover the entire posterior segment, potentially explaining why the MH did not close. Based on a study conducted on 57 eyes in 2006, Chen et al. [25] proposed that tamponade makes it difficult to overcome the posterior staphyloma curvature. Therefore, it reduces the retinal connection and results in failure to close or reopen the hole. Suda et al. [18] and Wu and Kung [19] suggested that owing to the presence of posterior scleral staphyloma, additional reverse vertical traction acts on the retina and hinders MH closure. Additionally, the latter considered that posterior scleral staphyloma increases the difficulty of ILMP. Consequently, a small amount of ILM and vitreous body remain after operation. Thus, the tangential traction force is not completely removed, thereby affecting the hiatus closure.

#### 2.3.3. Histopathological Changes of ILM

In 2018, Chen's [26] study showed that the HM group exhibited a markedly reduced ILM thickness, which may lead to difficulty in ILM peeling [27].

Alterations of the ILM thickness in HM eyes were accompanied by morphologic changes. The basic framework of the ILM is composed of networks of collagen, glial cells, and polymerized laminins. However, in high myopic patients, ILMs lost their homogeneous spongy meshwork. Also, expression levels of collagens and distributions of glial cells altered, which might influence the biomechanical properties of the ILMs.

In Chen's study, the number of astrocytes in the HM group was higher than that in the IMH group. Meanwhile, Yokota [28] indicated that the cellular elements and collagen appeared to have migrated through the ILM and adhered to the posterior vitreous cortex. Both facilitate the development of tangential traction at the vitreoretinal interface, which may cause closure failures.

#### 2.3.4. Chorioretinal Atrophy

In addition to axial elongation, posterior staphyloma and chorioretinal atrophy are marked pathological alterations associated with high myopia. Wu and Kung [19] considered that chorioretinal atrophy might cause difficulty in performing ILMP, resulting in remnant tangential traction force preventing MH closure. Chen et al. [25] and Hong et al. [29] agreed that chorioretinal atrophy is associated with poor retinal adhesion to RPE, increasing the possibility of retinal detachment producing additional traction force. Nevertheless, further studies on the relationship between chorioretinal atrophy and the prognosis of MH are warranted.

#### 2.3.5. Retinoschisis

In 2011, Suda et al. [18] found a retinoschisis-like feature in patients with highly myopic MH. Most of these changes occurred in eyes with an axial length ≥30.0 mm, which is highly related to the failure of anatomic closure. Suda et al. suggested that this feature may predict the anatomic failure of MH surgery in highly myopic eyes. Ohsugi et al. [30] divided MHs into two types (eyes with and without retinoschisis) and suggested that retinoschisis is a precursor of retinal detachment. In 2012, Jo et al. [31] compared patients with MHs + retinoschisis and those with simple MHs and reported that axial lengths were similar between groups. After the same operation, the closure rate of those with retinoschisis was lower than that of those with simple MHs. Alkabes et al. [32] proposed that inner retinal shortening is a major cause of the failures. In MHs with retinoschisis, retinal reattachment itself forces the inner retina to follow a larger arc made by the choroid/sclera, which may enlarge the MH postoperatively. However, further evidence and studies are required.

### 2.4. Traumatic MH

Traumatic MH (TMH) is a retinal hole in the fovea of the macula caused by a direct or indirect, closed, or open external force to the eyeball. Blunt-force trauma is the most common cause of TMH. Histologically, it is a partial or total tissue defect affecting the region from the inner limiting membrane to the photoreceptor cell layer. The pathogenesis of TMH remains unclear. Karaca et al. [33] proposed that TMH may occur because of the direct transmission of external forces, which results in the separation and traction of the attached vitreous body, leading to the direct rupture of the macula. Johnson et al. [34] reported that trauma causes sudden axial compression of the eyeball, reducing the anterior and posterior diameters of the eyeball. The eyeball expands in a compensable manner toward the equator because its volume remains unchanged. This structural change in the eyeball leads to the separation of the retinal layer at the fovea.

Since TMH often occurs in young and middle-aged patients, they show strong tissue repair, proliferation, and healing abilities. They also show a high rate of spontaneous healing. Gao et al. [35] conducted a meta-analysis of 12 studies and reported that in patients aged <24 years and with a hiatus aperture <0.2 times the optic disc diameter (particularly without other severe ocular trauma), spontaneous closure of the TMH occurs more. Miller et al. [36] suggested that patients should be observed for 2-3 months, and surgery should be performed if no spontaneous healing is observed. Spontaneous healing may occur after 3 months, but the probability is low. However, if the operation is performed ≥3 months after trauma, the closure rate of the hiatus may decrease.

To date, the standard procedure used for the treatment of IMH is also widely used in the surgical treatment of TMH. However, the prognosis of surgical treatments varies. Among 11 patients with TMH enrolled by Miller et al. [36], only five showed postoperative closure. Gao et al. [35] evaluated the data of 227 patients with TMH from six studies and showed that the postoperative hole closure rate ranged from 86.1% to 95.4%. Tang et al. [37] reviewed studies involving ≥10 patients with TMH; the postoperative closure rate after the first operation ranged from 46% to 100%. This discrepancy in the closure rate may be attributed to the complexity of ocular trauma. TMH may be accompanied by severe fundus lesions such as macular edema, retinal fold, pigment epithelium damage, and retinal choroid injury. Additionally, hole sizes were different. Huang et al. [38] compared OCT findings between patients with IMH and TMH. They found that the latter patient group showed lower mean retinal thickness, larger basal diameter and hole areas, and more irregular shapes than the former group. Predicting the outcome of vitreous surgery is arduous because of the diversity of TMH [39]. Strong adhesion between the vitreous body and the retina may be observed because patients are generally younger. This type of adhesion may cause the residual vitreous body to adhere to the retina and optic disc after the operation, leading to an incomplete release of traction force. Additionally, tight adhesions may lead to iatrogenic retinal tears and other complications. Johnson et al. [34] found that damage to the RPE is a significant manifestation of trauma. If there is an obvious rupture of the RPE near or within the fovea, it may affect the closure of the hole.

### 2.5. Macular Telangiectasia Type 2

Macular telangiectasia (MacTel) is a rare disease affecting the vasculature and architecture of the perifoveal retina. MacTel type 2 is often characterized by a gradual loss of vision, including metamorphopsia and scotoma.

Telangiectasia-induced complications in MH are rare. In 2006, Olson and Mandava [40] were the first to report such complications. Charbel Issa et al. [41] reported one complication in six patients, two of whom underwent vitrectomy. One patient was diagnosed with ERM-associated MH and telangiectasia. The hole remained open postoperatively. Another patient underwent vitrectomy and silicone oil tamponade, but the hole was not completely closed. It is suggested that in the presence of telangiectasia, MHs may have pathophysiological characteristics different from those of typical ones. In 2014, Karth et al. [42] conducted postoperative follow-up in four patients, and only one patient's MH remained closed. Recently, Miller et al. [43] reported that 4 of 12 eyes had a closure of the MH associated with MacTel type 2. Koizumi et al. [44] suggested that Müller cells provide the main structural support to the fovea. However, in telangiectasia, degeneration and loss of Müller cells lead to the formation of cavities in the foveal tissue. Charbel Issa et al. [45] confirmed the hypothesis that changes in Müller cells led to the destruction of the foveal structure. Therefore, the surgical closure rate was lower than that of IMH. In conclusion, macular telangiectasia, a rare complication, is associated with a lower anatomical success rate than IMH due to the loss of Müller cells in the fovea.

## 3. Surgical Factors

### 3.1. Treatment of ILM

#### 3.1.1. Standard (Traditional) ILMP

The ILM, which is the innermost layer of the retina, is a thin film between the retina and the vitreous body. It is the basement membrane in Müller cells, whose functions may involve maintaining the integrity of the retinal structure and preventing pathological migration of glial cells. In MHs of the third and fourth Gass stages, it is speculated that Müller cells and glial cells migrate to the ILM, transform into fibroblasts on the surface of the macular fovea, and even form the ERM. Their contraction produces tangential traction on the hole. Therefore, ILMP plays an important role in the anatomical healing of MHs. The ILM acts as a scaffold for the proliferation of RPE cells and myofibroblasts that produces a tangential traction force, resulting in poor closure rates and even enlargement and recurrence of the hole postoperatively [46]. However, after ILMP (including the epimembrane and residual vitreous body), the traction may be completely removed, potentially reducing retinal stiffness, restoring the retina ductility, and stimulating the proliferation of glial cells, thus facilitating the closure of the hole [47].

In 2016, a meta-analysis of 5,497 eyes from 50 studies by Rahimy and McCannel [48] showed that ILMP reduces the hole recurrence rate from 7.12% to 1.18%. Spiteri Cornish et al. [49, 50] showed that the ILMP improves the closure rate of holes and reduces the probability of reoperation. Moreover, it has no adverse effects on the complication rate. Some scholars believe that the ERM grows on the surface of the ILM but not on the cell membrane of Müller cells [51]. This suggests that residual ILM provides a scaffold for the growth of the ERM, and the ERM may prevent the holes from closing or even lead to their reopening (see Section 4.2.2). The exaggerated range of ILMP may cause retinopathy, such as retinal nerve fiber layer hemorrhage; thus, the range of ILMP becomes essential. Modi et al. [52] compared removal diameters of 3 mm and 5 mm and found no significant difference in the MH closure rate. However, Goker et al. [53] close as possible to the vascular arch in the macular region; otherwise, the residual ILM still has tangential traction, hindering MH closure. Yao et al. [54] considered that when MHCI is <0.5, the anatomic prognosis of patients with enlarged ILMP (4 optic disc diameters) was better than that of patients with a small ILMP (2 optic disc diameters). Furthermore, in the second operation of patients with postoperative patent holes, complete hole closure occurred after expanding the range of ILMP. Wang and Wang [55] considered the diameter of the ILMP as an important factor affecting the hole closure. The traction and adhesion of the posterior retina increased while the axial length increased. Therefore, in patients with highly myopic MHs, ILMP on a small area will lead to a decrease in the MH closure rate. Wang and Wang performed ILMP in a centripetal direction to avoid the formation of an MH and extended the scope of the ILMP to the temporal vascular arch.

#### 3.1.2. Hole Reopening after ILMP

Some scholars believe that iatrogenic injury to the retina during surgery increases postoperative recurrence [56]. Rubinstein et al. [57] were the first to report the appearance of iatrogenic MHs after surgery and suggested that they appeared because of the ILM-induced trauma. They also proposed that bleeding occurring in a small area during peeling may form holes after surgery. Hussain and Mitra [58] reported that the detachment of the ILM may cause mechanical damage to the inner retina, which may lead to apoptosis of glial cells and degeneration of neurons and result in the formation of postoperative extrafoveal MHs. Steven et al. [59] found that glial cell apoptosis may be caused by injury to the Müller cell endplate. Consequently, changes in retinal glial structure may occur. Such damage and changes may activate the regeneration mechanism, induce the proliferation of glial cells, and facilitate hole closure; however, they may also weaken the central glial structure of the retina, thus forming new holes. Moreover, Brouzas et al. [60] found that ILMP damaged Müller cells. The main function of Müller cells is maintaining the stability of the retinal nerve epithelium. The damage and apoptosis of Müller cells cause apoptosis of photoreceptor cells and full-thickness retinal defects. Some scholars speculated that the area of ILMP may need to be limited because an iatrogenic hiatus affected the scope of ILMP [59].

#### 3.1.3. Improvements and Changes in ILMP

In 2014, Ho et al. [61] proposed that it is feasible to preserve the ILM of the fovea in patients with stage II IMH by performing donut-shaped ILMP, leaving an ILM with a diameter of 400 *μ*m. Ho et al. [62] explained that the foveolar ILM nonpeeling technique, better than ILMP, ensures that the tissue remains securely in position. Besides, tractions from 360 degrees around can be released completely and evenly so as to achieve a symmetric foveolar architecture.

However, the sample size of this operation was small and required further follow-up and research.

*(1) Inverted ILM Flap Technique*. To reduce the recurrence rate of ILMP-induced MHs, Michalewska et al. [63] proposed an inverted ILM flap technique. They peeled the ILM of approximately two optic disc diameters to the edge of the MH in a circular manner around the MH, leaving a remnant attached to the margins of the MH. This ILM remnant was then inverted upside down to cover the MH. For some refractory types of MHs, particularly large MHs, the healing rate of a simple ILMP is low. Moreover, there is a risk of reopening. This technique may effectively improve the closure rate of large MHs and reduce the occurrence of iatrogenic injuries caused by extensive ILMP. Hu et al. [64] peeled off a 200–300-*μ*m wide annular ILM around the hole and created a tongue-like flap on the upper part of the retina. The inferior edge of the flap was not peeled off. The edge of the flap was grasped, and the hole was covered upside down. This technique reduces the incidence of side effects by reducing the range of the ILMP and avoiding or reducing the damage caused by ILMP to the retinal microstructure. Finally, it provides a scaffold for healing and helps to achieve an improved rate of hole closure. In Hu's study [27], the myopic MH closure rate 100% versus 66.7% was significantly better in the eyes treated with the inverted ILM flap technique than those treated with the ILM peeling technique.

Rizzo et al. [65] compared the hole closure rate of patients with simple ILMP and inverted ILM flaps. They found that, in some patients with refractory MHs, the anatomical prognosis of simple ILMP is not ideal, irrespective of whether the hole diameter or axial length is ≥400 *μ*m or ≥26 *μ*m, respectively. The hole closure rate of the inverted ILM flap technique is significantly higher than that of ILMP. Yamashita et al. [66] divided 165 patients with an MH diameter >400 *μ*m into two groups: patients with diameters >550 *μ*m and those with <550 *μ*m. They reported that although no statistically significant difference is observed owing to the small sample size, a simple ILMP yields a lower closure rate when a hole is very large compared with cases where it is medium-sized. Regardless of the hole size, the inverted ILM flap achieved a 100% closure rate. Therefore, for MHs with a diameter >550 *μ*m, the inverted ILM flap technique seems a reasonable choice to improve the closure rate. Shiode et al. [67] found that the ILM flap covering the hole may be used as a scaffold for glial cell proliferation. Moreover, it may close the hole, prevent the fluid in the vitreous cavity from entering the hiatus, and promote the closure of the hiatus. Additionally, several studies have demonstrated that the inverted ILM flap may improve the closure rate of the hiatus [68, 69]. Furthermore, a number of ILM flap techniques have emerged, achieving better closure rates regarding the treatment of large MHs and high myopic MHs, which are difficult to manage by standard ILMP [70].

*(2) ILM Packing*. For an MH in which ILM has been peeled or that has a secondary hole after operation, the closure rate with the existing operation method is low. Therefore, Morizane et al. [71] used autologous transplantation of ILM. They transplanted the free ILM into the hole and placed a low-molecular-weight viscous material on it to fix the free flap. This method was proven to be effective for the treatment of refractory MH, even in patients with high myopia whose MH cannot be closed using traditional ILMP. Chen and Yang [72] found that the ILM flap may be detached from the hole during or after operation, potentially hindering hole closure. Therefore, Chen and Yang peeled the ILM in a circular way, leaving a ring-shaped ILM island of approximately 1.5–3 disc diameters centered on the MH. The ILM around the hole was then detached from the retina up to the edge of the hole (the edge of the hiatus was not broken), which was inverted and inserted into the hole. They believe that this method may prevent the flap from falling off, facilitating improved bridging and closure of the MH. Finally, they confirmed that ILM insertion yields better MH closure rates in patients with high myopia than simple ILMP. In 2018, Wakabayashi et al. [73] conducted a 12-month follow-up of 49 patients with MH complicated by retinal detachment and found that the patients who underwent ILM insertion achieved a higher MH closure rate than those who underwent standard ILMP. In a meta-analysis including 151 cases of MH with high myopia from five studies, the standard ILMP and ILM insertion were compared. ILM insertion showed a higher hole closure rate [74].

Additionally, in 2017, Rossi et al. [75] compared the inverted ILM flap technique and ILM insertion and reported no statistically significant difference in the closure rate yielded by the two surgical methods at 6 months after operation for holes with diameters slightly larger than 400 *μ*m. However, for larger holes, they believed that ILM insertion may be more effective than the inverted ILM flap method. Rossi et al. considered that the inserting flap is more conducive to the closure of the holes than the inverted flap technique because it plays the role of filling, glue, and proliferating scaffold.

### 3.2. Selection of Dye

The ILM is a transparent thin film, which makes it difficult to distinguish the boundary and scope of stripping. Therefore, peeling it off is challenging. However, the use of a dye makes ILMP manageable, controllable, and safe. To date, the commonly used dyes include indocyanine green (ICG), brilliant blue G (BBG), trypan blue, and triamcinolone acetonide (TA). Wu et al. [76] performed a meta-analysis including 1,585 eyes from 22 studies and found no significant differences in the anatomical reduction rate between ICG and non-ICG staining. Azuma et al. [77] considered that BBG staining was safer than ICG staining. Although the clarity was poor, no significant difference was observed in the closure rate of the hole after surgery. These studies showed that most dyes effectively stain the ILM. This helps surgeons remove the ILM cleanly and thoroughly, facilitating hole closure. However, although TA attaches itself to the residual posterior vitreous cortex, the effect is not ideal because it cannot stain the ILM. Additionally, some studies [78, 79] reported that TA may delay and hinder hole closure. Both reports suggest that this may be attributable to a mechanical block by the TA crystals of the physiological interactions between the sensory retina and the retinal pigment epithelium (RPE). Alternatively, it may have been caused by the effect of TA to alter the function of RPE cells, resulting in failure of closure.

Most recently, Sen et al. [80] raised the opposite opinion. They thought that apart from the anti-inflammatory role which brought down the retinal edema, TA acts as a temporary tamponade. The TA plugs the foveal defect and prevents further movement of vitreous fluid into the subretinal space, which helps the holes close.

### 3.3. Tamponade

Intravitreal tamponade is often required after surgery, providing a scaffold for the migration and proliferation of RPE. It also maintains the eye's volume and pressure, closes the hole, and prevents the liquefied vitreous body from entering the retina. Commonly used fillers include an inert gas, disinfected air, and silicone oil.

Silicone oil cannot be absorbed by the human body and requires removal by a secondary surgery. Moreover, it has numerous side effects, while its particles penetrate the optic nerve and cause nerve damage [81]. Additionally, Lai et al. [82] reported that silicone oil shows a slightly lower closure rate than inert gas, while inert gas tamponade may promote glial cell responses. Furthermore, the buoyancy of gas is higher than that of silicone oil, thus fixing the hole edge more effectively and pushing the retina to the RPE. Finally, it increases the isolation effect between the hole and the liquefied vitreous, thereby promoting closure.

The absorption of various gases is faster than that of silicone oil. Furthermore, the use of various gases requires no secondary surgery and is associated with few side effects. Essex et al. [2] and Modi et al. [83], among others, found no statistically significant differences in the closure rates of MHs among different inert gases. In 2017, Hou [84] showed that the use of inert gas or disinfected air did not affect the closure rate of MHs (51 eyes) with a diameter <600 *μ*m. A retrospective study (300 eyes) conducted in 2019 [85] found that the closure rate achieved using sterilized air was significantly lower than that using an inert gas when MH diameters were ≥650 *μ*m. Additionally, the hole closure rate achieved using disinfected air decreased while the hole diameter increased when the latter was ≥650 *μ*m. This trend may be attributed to the short half-life of air and insufficient top pressure.

Some scholars believe that [86] the filling degree of gas tamponade is related to postoperative hole closure. When gas tamponade is insufficient, the top pressure of the macula is also insufficient, potentially leading to hole closure failure. The risk of surgical failure was reduced when the gas volume was >65% on the 4^th^ postoperative day. Alberti and La Cour [86] attributed this to insufficient gas filling at the first time, potentially resulting in poor gas contact with the macula and failure to close the hole.

## 4. Postoperative Factors

### 4.1. Postoperative Positioning

According to the traditional view, with regard to the postoperative effects of tamponade, the face-down position may maximize the sagittal traction force pointing toward the hole and ensure that the tamponade material fully tops the fracture hole edge and facilitates hole closure.

However, maintenance of a prone position affects the long-term physical and mental state. With the expansion of OCT, numerous studies reported that hole closure may occur within minimum 1 postoperative day; thus, the duration for which the postoperative prone position must be maintained is shortened [87]. Alberti and La Cour [86] considered that the prone position is unnecessary for most MHs. However, the authors also acknowledge that in the nonprone group, closure failure may occur because of postural factors, particularly in eyes with insufficient gas filling. Several studies have shown that the postoperative effect of the prone position is related to the aperture of the holes; supporting the prone position for a long time is unnecessary when MHs are small. However, it is absolutely necessary when MHs are large [46]. According to a meta-analysis by Hu et al. [88] in 2016, when an MH is small or slightly larger than 400 *μ*m, postoperative positioning does not affect hole closure. However, the prone position is of greater significance when MHs are large. Some scholars believe that OCT should be used to evaluate whether the hole is closed, to guide patients on the duration of maintaining a prone position [89]. Chow and Chaudhary [90] reported that guidance on the requirement of prone position should be provided according to OCT-based findings and assessed according to the presence of risk factors. If the patient presents no risk factors (duration of MH > 1 year, high myopia, hole diameter >400 *μ*m), the maintenance of a prone position can be stopped after hole closure evaluation using OCT. If the patient presents any risk factors, maintenance of a prone position may be extended for 2-3 days after hole closure. For multiple risk factors, the prone position should be maintained for 7 days. The authors found that most hiatus is closed within 1 postoperative day, but there are risk factors. If the patient cannot ensure adequate prone time, there may be a risk of anatomical failure.

### 4.2. Retinal-Related Changes

#### 4.2.1. Cystoid Macular Edema (CME)

CME may be a risk factor for failure of MH closure.

The hydration theory by Tornambe [91] explains why macular holes disappear after successful surgery: fluid is pumped out of the macula, the “drawbridge” closes, and the edges of the fovea reapproximate, which means the critical factors for hole closure are relieving traction and isolating the hole from the vitreous fluid. However, in holes with CME, degeneration and atrophy of the outer retina develops in the swollen area, which keeps the retracted inner retina from approximating. Furthermore, CME creates extra tension. Both of these will hinder the closure of the holes. Additionally, according to Gentile et al. [92], the development of an MH is accompanied by cystic thickening of the central fovea. The diameter of the IMH is related to the formation of the perifoveal cystic cavity. In contrast, the formation and expansion of cystic edema may lead to the enlargement of the diameter of the hole. Therefore, this may hinder the closure of the MH postoperatively.

In 2000, Paques et al. [93] proposed that any process that disturbs the normal foveolar anatomy, including the development of CME, may lead to the reopening of macular holes. An CME may elevate the edge of the inner retinal defect, and then the hole may reform. Lee et al. [94] reported that a CME-related mechanism is involved in a major type of postoperative MH reopening. After surgery, small cysts merge to form larger ones, which may rupture or degenerate to form a hole. This may eventually lead to the reopening of the MH. CME may also be an indirect cause of reopening, because the healing process contributing to the hole closure is impaired by histologic modifications.

#### 4.2.2. ERM

The ERM is among the main causes of MH recurrence. Lee et al. [94] reported that the tangential traction force produced by the ERM acts on the fovea, potentially leading to the development of subfoveal cysts and then full-thickness MHs. Paques et al. [95] found an ERM in 4 out of 5 patients with recurrent MH. In their retrospective controlled study, Yoshida et al. [51] found that ERMs developed in all recurrent MHs after surgery, and all of them occurred when the holes opened again. They suggested that the tangential traction force produced by the ERM caused the recurrence of MHs. The formation of ERMs may be reduced or even eliminated by ILMP. However, there is scant literature on the development of the ERM after ILMP. In addition to the residual ILM and ERM contracture, which may produce traction after ILMP [55], Uemoto et al. [96] considered that endplates of Müller cells are damaged by ILMP. These promote the proliferation of glial cells and their migration to the macular surface through the injury-induced gap, thus leading to the formation of the ERM.

#### 4.2.3. Retinal Pigment Epithelium

According to the hydration theory proposed by Tornambe in 2003 [91], closure of postoperative holes occurs because of the isolation of the liquefied vitreous body from the MH by surgery and gas tamponade. Thus, the vitreous fluid cannot continue to pass through the hole or penetrate the retina. Moreover, the RPE continuously pumps out the inner retinal cyst fluid and subretinal fluid and finally restores the original macular structure. Therefore, Tornambe suggested that, in patients with diseases involving RPE damage, such as age-related macular degeneration, the pumping function is reduced or disappears. Consequently, the fluid in the retina is not pumped out, and the anatomical structure is not restored. Therefore, the holes are not closed.

### 4.3. Cataract Surgery

Cataract is among the most common complications after vitrectomy and is also a risk factor for postoperative MH recurrence. Ameli and Lashkari [97] reported that CME after cataract extraction is the main mechanism of hole recurrence. In the late stage of recurrence, the formation of the ERM may further accelerate its development. Bhatnagar et al. [98] compared the classification of patients according to the order of cataract extraction and vitrectomy. They concluded that cataract extraction after vitrectomy increases the risk of hole recurrence. Bhatnagar et al. reported that CME is a complication potentially leading to MH reopening. In patients who underwent cataract extraction after MH surgery, the risk of CME hole recurrence with significant clinical characteristics is increased by seven times. Additionally, the promoting mechanism of inflammation and fibrinolysis also advances the reopening of the hole. After cataract extraction, the direct mechanical action and inflammatory reaction during the operation contribute to fibrinolysis, thereby reducing the adhesion between the glia and retina. Therefore, the continuous remodeling of cells of the retina and in front of the retina and the resulting traction force loosen the connection between the glial cells [99].

## 5. Conclusions

Recent improvements in the techniques of ophthalmic examination and surgery have advanced research on MH. Vitrectomy combined with ILMP is currently the primary method used to treat MHs. The closure rate of MHs may reach >90%. However, MH closure fails in some patients owing to the course and characteristics of MH, presence of high myopia and other complications, intraoperative operation, and postoperative posture among others. Meanwhile, macular hole reopening after successful surgical repair is well documented, and it is commonly caused by cataract surgery, growth of an ERM, and development of CME. Based on the etiology and operation process, we summarized the factors that may cause failure and recurrence to facilitate a more accurate outcome prediction. This will help doctors determine whether there are risk factors for postoperative anatomic failure and develop appropriate treatment methods. Furthermore, it may provide a basis for future surgical improvements to achieve a higher hole closure rate.
